# Comparison of two hyaluronic acid formulations for safety and efficacy (CHASE) study in knee osteoarthritis: a multicenter, randomized, double-blind, 26-week non-inferiority trial comparing Durolane to Artz

**DOI:** 10.1186/s13075-015-0557-x

**Published:** 2015-03-10

**Authors:** Heng Zhang, Ke Zhang, Xianlong Zhang, Zhenan Zhu, Shigui Yan, Tiansheng Sun, Ai Guo, John Jones, R Grant Steen, Bin Shan, Jenny Zhang, Jianhao Lin

**Affiliations:** Arthritis Clinic & Research Center, Peking University People’s Hospital, 11 Xizhimen South Street, Xicheng District, Beijing, 100044 China; Department of Orthopedics, Peking University Third Hospital, Beijing, China; Department of Orthopedics, Shanghai 6th People’s Hospital, Shanghai, China; Department of Orthopedics, Shanghai 9th People’s Hospital, Shanghai, China; Department of Orthopedics, The Second Affiliated Hospital of Zhejiang University School of Medicine, Zhejiang Province, China; Department of Orthopedics, Beijing Military General Hospital, Beijing, China; Department of Orthopedics, Beijing Friendship Hospital, Beijing, China; Bioventus LLC, Durham, NC USA; TigerMed Consulting Co., Ltd, Beijing, China

## Abstract

**Introduction:**

Intra-articular injection of hyaluronic acid (HA) is often used as therapy for knee osteoarthritis because it is less expensive and less aggressive than total knee replacement. Therefore, it is important to document whether HA is safe and efficacious. We tested whether single and multiple injection viscosupplementation with HA is associated with clinically meaningful pain relief in a new randomized clinical trial (RCT). Our objective was to compare safety and efficacy of intra-articular HA in two formulations: one 3.0 ml injection of Durolane versus five 2.5 ml injections of Artz for the treatment of knee osteoarthritis pain.

**Methods:**

Patients (*N* = 349) from the People’s Republic of China were randomized to treatment (Durolane = 175, Artz = 174). The Durolane group received a 3.0 ml injection at week 0 (baseline), with sham skin punctures at weeks 1, 2, 3, and 4. The Artz group received one 2.5 ml injection at each of the same time points. The primary assessment tool was the Likert-type Western Ontario and McMaster University (WOMAC) pain scale at weeks 0, 6, 10, 14, 18, and 26. Secondary assessments were WOMAC physical function, knee stiffness, and global self-assessment, at identical time points. Statistically-controlled analyses were non-inferiority of Durolane over 18, then over 26 weeks, with *a priori* non-inferiority defined as 8% of the relevant scale. Acetaminophen was permitted as rescue analgesia and all adverse events (AEs) were recorded.

**Results:**

Overall study retention was excellent; 332 patients (95.1%) completed 18 weeks and 319 (91.4%) completed 26 weeks, with no significant retention difference between treatment arms. All variables met non-inferiority criteria over 18 and 26 weeks. Efficacy response in both arms was >90%. Treatment-related AEs were 9.8% (17/174) for Artz and 13.1% (23/175) for Durolane.

**Conclusions:**

A single injection of Durolane is non-inferior to 5 injections of Artz over 18 and 26 weeks for pain, physical function, global self-assessment, and knee stiffness. Both treatments were efficacious, safe, and well tolerated.

**Trial registration:**

ClinicalTrials.gov NCT01295580. Registered 11 February 2011.

## Introduction

Knee osteoarthritis (OA) is a degenerative joint disease that caused moderate to severe disability in 43.4 million people globally in 2004 [1]. Aggregate economic costs are considerable because treatment is not curative [2]; current treatment focuses on relieving pain and other symptoms, as well as improving function [3,4].

Intra-articular injection of hyaluronic acid (HA) is widely used as therapy because: it is less aggressive [5] and less expensive [5-9] than total knee replacement; HA has fewer and generally less serious adverse events (AEs) than total knee replacement [10,11]; and HA is documented to provide treatment efficacy [12-19]. Nevertheless, a recent meta-analysis concluded that ‘In patients with knee osteoarthritis, viscosupplementation is associated with a small and clinically irrelevant [pain] benefit and an increased risk for serious adverse events’ [20]. We seek to test whether single-injection and multiple-injection viscosupplementation is associated with clinically meaningful pain relief in a new randomized clinical trial (RCT).

Our goal was to test for non-inferiority of two formulations of HA in treatment of knee OA over an 18-week and then over a 26-week time period. We contrast Durolane (Q-med AB, Sweden), a stabilized HA that is obtained from a nonanimal source, with Artz (also marketed under the names Artzal and Supartz; Seikagaku Corporation Japan), a noncross-linked (native) animal-derived HA that has been available on the Chinese market since 1997.

## Methods

### Overview

We report a multicenter, randomized, double-blind, 26-week non-inferiority trial comparing intra-articular injection of Durolane (one dose plus four sham injections) with intra-articular Artz (five doses) in the treatment of knee pain among adults clinically diagnosed with mild-to-moderate knee OA.

The institutional review board or ethics committee for each study site (see Acknowledgements) was responsible for review and approval of the clinical study, in accordance with guidelines of the International Conference on Harmonization, as well as any local regulatory requirements of each site. Written approval of the protocol, amendments, and the patient informed consent form were submitted to Bioventus LLC (Durham, NC, USA) before study drugs were shipped to each site.

Subjects were recruited consecutively at any of seven hospitals between January 2011 and February 2012, a period of approximately 1 year. Each potential subject provided written informed consent and underwent a qualifying screening examination prior to study enrollment. Each patient consented that their study data could be examined by the sponsor, drug regulatory authorities, auditors, and study monitors, in compliance with the statement of confidentiality.

After consent, eligible subjects were allocated randomly to either Durolane or Artz. Randomization was double-blinded, using a 1:1 allocation in block sizes of four patients. The allocation schedule was generated by statisticians at TigerMed Consulting Co., Ltd (Beijing, China), using SAS Proc Plan (v9.1.3; Cary, NC).

### Patients

Patients were recruited for the study from seven sites in the People’s Republic of China (Beijing, four hospitals; Shanghai, two hospitals; Hangzhou, one hospital). Assessment at screening included a radiograph of the study knee in the standing, weight-bearing, semi-flexed, postero-anterior view, as assessed by the X-ray reader assigned to each study center. Inclusion criteria were as follows: males or females, age 40 to 80 years; physician diagnosis of mild-to-moderate OA fulfilling American College of Rheumatology criteria [21], as recorded in the chart; radiographic evidence of OA (Kellgren–Lawrence radiographic score of 2 or 3); Western Ontario and McMaster University (WOMAC) Likert pain subscore of 7 to 17 at both screening and baseline visits; and WOMAC Likert pain question score of 2 to 3 while walking on a flat surface.

Patient exclusion criteria were: clinically apparent tense effusion on physician examination, determined by either a positive bulge sign or positive ballottement of the patella; Kellgren-Lawrence radiographic score of 0, 1, or 4 in the study knee; symptomatic OA of the contralateral knee (or of either hip) that is not responsive to acetaminophen and/or that requires any change in physical therapy; WOMAC Likert pain question score >3 in the contralateral (nonstudied) knee; intra-articular injection of any HA in the study knee within 9 months prior to screening; previous allergic reaction to any HA product; intra-articular injection of corticosteroids in any joint within 6 months prior to screening; treatment with glucosamine–chondroitin sulfate supplement initiated within the past 3 months, or dosage not stable for the past 3 months; active skin disease or infection at the injection site; active hepatic disease, abnormal liver function (Alanine transaminase, Aspartate transaminase or Total bilirubin level more than twice normal values), or renal dysfunction (blood creatinine over the upper limit of normal); systemic inflammatory conditions, autoimmune diseases, connective tissue diseases (including rheumatoid arthritis, inflammatory arthritis, ankylosing spondylitis, psoriatic arthritis, reactive arthritis, gout/acute pseudo-gout), or uncontrolled hypothyroidism; bleeding diathesis or use of anticoagulants (except aspirin <325 mg/day); and any other medical condition that might make the patient unsuitable for study (for example, any musculoskeletal condition impeding measurement of efficacy at the studied knee, severe progressive chronic disease, malignancy, bleeding disorder, fibromyalgia, significant venous or lymphatic stasis).

### Treatment

Treatment procedures for both products were identical. After screening, patients were given a 2-week washout period for analgesics other than acetaminophen. At week 0, patients were randomized 1:1 and received their first injection of either Durolane or Artz. Injections followed at weeks 1, 2, 3, and 4. For the Durolane arm, all subsequent injections were sham injections; for the Artz arm, all injections were active. Sham injections of Durolane were subcutaneous and used an empty syringe, and the needle did not enter the joint space. Subcutaneous sham needles have proven indistinguishable from deeper needles in clinical trials of acupuncture [22].

Disinfectants containing quaternary ammonium salts such as benzalkonium chloride, which can induce HA precipitation, were avoided. Anesthetization of the injection site was permitted using a topical anesthetic (for example, ethyl chloride or lidocaine spray).

Patient blinding was achieved by draping, so that patients could not determine by sight how the injection was given or what product was injected. Patients were also told that each injection could feel different, to minimize the placebo effect in the Durolane arm. Physicians who gave injections could not be blinded, but outcome assessment was done by different physicians blind to the study treatment.

Physicians were allowed to inject HA at the knee portal with which they were most experienced (lateral upper patellar, lateral mid patellar, or medial mid patellar). Needles (sizes 20 G and 22 G) were supplied to each study site and unblinded personnel chose the appropriate needle. Joint fluid was withdrawn using an empty 20 ml syringe and the volume of aspirated fluid was recorded. Leaving the needle in place, the syringe was removed and replaced by a prefilled Durolane or Artz syringe. Care was taken when exchanging syringes to avoid displacement of the needle and to ensure that the syringe with the study product was securely attached prior to injection.

### Efficacy assessments

The only permitted rescue medication for pain was acetaminophen, at doses up to 4 g daily. Efficacy assessments were collected at weeks 0 (baseline), 6, 10, 14, 18, and 26. The 18-week follow-up period was the primary efficacy assessment period. Efficacy assessments included the Likert-type WOMAC pain scale (range 0 to 20, higher scores better), with physical function (range 0 to 68) and knee function (range 0 to 8) subscales [23]. The WOMAC translation into Chinese has been validated in Chinese patients [24]. Global self-assessment scores (range 0 to 10, lower scores better) and total grams of acetaminophen used were also collected.

### Safety

AE reports were collected continuously, physical examinations and vital signs were assessed at week 0 and 26, and electrocardiograms and blood and urine laboratories were assessed at weeks 0 and 10.

### Statistical methods

Three datasets were defined for analysis. The intention-to-treat (ITT) set included all patients randomized. The safety set included all patients who received treatment. The per-protocol set included ITT patients who completed all scheduled treatments and also completed all WOMAC pain subscale assessments through week 18, without any major protocol deviations. The per-protocol set was used to assess non-inferiority; since all patients received all treatments and had minimal protocol deviations, this should maximize the difference between treatment arms, if a difference truly exists.

The primary efficacy variable was the WOMAC pain subscale change from baseline (CFB). Secondary efficacy variables were the WOMAC physical function subscale CFB, the global self-assessment CFB, and the WOMAC knee stiffness subscale CFB. All other efficacy variables assessed were exploratory, including the Outcome Measures in Rheumatoid Arthritis Clinical Trials – Osteoarthritis Research Society International (OMERACT-OARSI) responder variable [25] and use of rescue medications. Exploratory subgroups were split by gender, age (cutoff point >50 years old), and Kellgren–Lawrence grade II and III at baseline.

Important AEs resulted in a dose adjustment, interruption, or permanent stop. AEs were defined as treatment-emergent adverse events (TEAEs) or treatment-related adverse events (TRAEs). TEAEs included all medical events reported after the first treatment. TRAEs were judged by the site investigator to have a definite, possible, or uncertain relationship to treatment. Both TEAEs and TRAEs were reported, including events classified by the investigator as possibly, probably, or definitely treatment related. Vital signs, blood, and chemistry laboratories were used to study the CFB. Laboratory values and electrocardiograms were also used to study shifts from baseline.

The required sample size was calculated under several assumptions for the WOMAC pain subscale; change standard deviation is 20 mm with a non-inferiority margin of +8 mm [26] (8% on the 100 mm visual analog scale), which is +1.6 on a Likert 0 to 20 scale, with a type I error rate of 0.05 and a type II error rate of 0.10. The required sample size under these assumptions was 132 per arm, for a total of 264 patients. To account for an anticipated 25% loss to follow-up and important protocol deviations, the recruited sample size was increased to 175 per arm, for a final total of 350 patients.

Although the study sample size was based on the WOMAC pain scale, we also evaluated WOMAC physical function, knee stiffness, and global self-assessment in a stepwise manner to control overall type I error at 0.05. Non-inferiority tests used 8% margins of the respective scale: pain, +1.60; physical function, +5.44; knee stiffness, +0.64; and global self-assessment, −0.80.

Non-inferiority hypotheses were tested using mixed-effects repeated-measures models. Primary and secondary variables were fitted to mixed-effects repeated-measures regressions, with subscale CFB the dependent variable. Subject baseline assessment, study site, treatment, visit, and treatment-by-visit interactions were fixed effects, patients were the random effect, and degrees of freedom were calculated by an established method [27]. Non-inferiority was concluded if the upper bound of the (Durolane – Artz) 95% confidence interval was less than the non-inferiority margin; or, in the case of the patient global self-evaluation, if the lower bound was greater than this margin.

Because non-inferiority hypothesis testing was carried out over two time periods (18 and 26 weeks) and for four assessments (a primary aim for pain and secondary aims for physical function, global self-assessment, and knee stiffness), it was necessary to predefine test order to control for overall type I error. The pain primary variable was first tested for non-inferiority over 18 weeks; if non-inferiority was concluded, then non-inferiority was tested again over 26 weeks. Physical function was then tested in the same manner, followed by subject global self-assessment, and finally knee stiffness. If any individual test failed to conclude non-inferiority, then subsequent hypothesis tests for non-inferiority would not qualify as controlled for type I error.

Exploratory responder variables were analyzed using generalized estimable equation regressions with corresponding explanatory variables. No formal type I error-controlled hypothesis testing was planned.

Study results were audited by TigerMed Consulting Co., Ltd, evaluating each research site and assessing compliance to the clinical trial protocol. Site visits included a random audit of patient records, and a study report was filed at the People’s Hospital of Beijing University and at Bioventus LLC. TigerMed Consulting Co., Ltd was also responsible for data management and statistical analysis.

## Results

**Figure 1:**
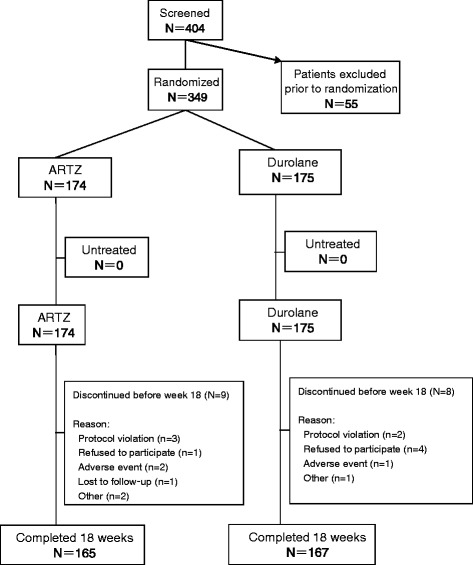
**CONSORT flow chart for subjects who were enrolled in the Comparison of Hyaluronic Acids for Safety and Efficacy (CHASE) Trial** **.**

A total of 404 patients were screened and 349 (86.4%) patients were randomized to treatment (Figure 1). Of these 349 ITT subjects, 319 (91.4%) qualified for the per-protocol set analysis. Baseline demographics and clinical characteristics (Table 1) were balanced between treatment arms. Overall, 97.8% of patients were Han Chinese and 75.2% of patients were naive to any treatment.

**Table 1:** **Demographics and baseline clinical characteristics (per-protocol set)**

	**Artz**	**Durolane**
	**(** ***n*** **= 158)**	**(** ***n*** **= 161)**
Age (years)		
Number	158	161
Mean (standard deviation)	60.4 (7.75)	60.2 (8.06)
Minimum; maximum	42; 78	40; 78
Sex (female)	127 (80.4)	119 (73.9)
Body mass index classification		
Underweight	0 (0.0)	1 (0.6)
Normal range	76 (48.1)	81 (50.3)
Overweight	69 (43.7)	65 (40.4)
Obese	13 (8.2)	14 (8.7)
Duration of disease (months)		
Number	157	161
Mean (standard deviation)	47.7 (57.59)	47.2 (63.15)
Median	22.2	17.6
ACR criteria		
Significant knee pain most days during the last 3 months	158 (100%)	161 (100%)
Age >50 years	147 (93.0%)	147 (91.3%)
Morning stiffness <30 minutes	129 (81.6%)	127 (78.9%)
Crepitus on motion	141 (89.2%)	148 (91.9%)
Surgical history		
Intra-articular steroid injection	5 (3.2%)	5 (3.1%)
Intra-articular hyaluronic acid injection	28 (17.7%)	36 (22.4%
Arthroscopy and/or other surgical procedure	8 (5.1%)	4 (2.5%)
No treatment	122 (77.2%)	118 (73.3%)
X-ray examination		
II: definite osteophyte, unimpaired joint space	95 (60.1%)	94 (58.4%)
III: moderate diminution of joint space	63 (39.9%)	67 (41.6%)
Likert-type WOMAC subscales^a^		
Pain (range 0 to 20)	9.5 (1.80)	9.4 (1.98)
Physical function (range 0 to 68)	22.7 (8.00)	22.3 (8.38)
Knee stiffness (range 0 to 8)	3.1 (1.77)	2.8 (1.73)
Global self-assessment^b^ (range 0 to 10)	4.3 (2.06)	4.3 (1.97)

Data presented as number, *n* (%) or mean (standard deviation) unless stated otherwise. Durolane from Q-med AB (Sweden) and Artz from Seikagaku Corporation (Japan). ACR, American College of Rheumatology; WOMAC, Western Ontario McMasters University Osteoarthritis Index. ^a^Lower scores better. ^b^Higher scores better.

The primary analysis over 18 weeks showed that Durolane was non-inferior to Artz in terms of pain control; this was also true over 26 weeks (Table 2). All secondary efficacy variables were tested over week 18 and week 26, and Durolane was non-inferior to Artz in all comparisons. Mixed-effects repeated-measures results are shown by week 18 and by week 26 (Table 2).

**Table 2:** **Non-inferiority variables: mixed-effects repeated-measures results, weeks 18 and 26 (per-protocol set)**

	**Change from baseline**		**Treatment-related**
	**Artz**	**Durolane**	**difference**
	**(5 × 2.5 ml)**	**(1 × 3 ml, 4 × sham)**	**(Durolane – Artz)**
	**(** ***n*** **= 158)**	**(** ***n*** **= 161)**	
**Pain** ^**a**^			
Week 18			
LSM (95% CI)	−5.87 (−6.23; −5.52)	−5.97 (−6.32; −5.61)	−0.09 (−0.58; 0.39)
*P* value	<0.0001	<0.0001	0.7034
Week 26			
LSM (95% CI)	−6.05 (−6.39; −5.71)	−6.15 (−6.49; −5.81)	−0.10 (−0.56; 0.37)
*P* value	<0.0001	<0.0001	0.6783
**Physical function** ^**a**^			
Week 18			
LSM (95% CI)	−12.10 (−12.95; −11.26)	−12.75 (−13.60; −11.91)	−0.65 (−1.81; 0.51)
*P* value	<0.0001	<0.0001	0.2718
Week 26			
LSM (95% CI)	−12.58 (−13.39; −11.77)	−13.16 (−13.97; −12.35)	−0.58 (−1.69; 0.53)
*P* value	<0.0001	<0.0001	0.3054
**Global evaluation** ^**b**^			
Week 18			
LSM (95% CI)	2.55 (2.33; 2.77)	2.70 (2.48; 2.92)	0.15 (−0.15; 0.45)
*P* value	<0.0001	<0.0001	0.3319
Week 26			
LSM (95% CI)	2.67 (2.45; 2.88)	2.81 (2.59; 3.02)	0.14 (−0.16; 0.43)
*P* value	<0.0001	<0.0001	0.3583
**Knee stiffness** ^**a**^			
Week 18			
LSM (95% CI)	−1.73 (−1.87; −1.59)	−1.87 (−2.00; −1.73)	−0.14 (−0.33; 0.05)
*P* value	<0.0001	<0.0001	0.1602
Week 26			
LSM (95% CI)	−1.80 (−1.93; −1.67)	−1.95 (−2.08; −1.82)	−0.15 (−0.33; 0.03)
*P* value	<0.0001	<0.0001	0.1012

Durolane from Q-med AB (Sweden) and Artz from Seikagaku Corporation (Japan). CI, confidence interval; LSM, ???. ^a^A negative change from baseline is an improvement. ^b^A positive change from baseline is an improvement.

Patients responded well to both Durolane and Artz (Table 3); however, there were twice as many nonresponders with Durolane in the WOMAC pain ‘walking on a flat surface’ variable. Rescue medication use was comparable between treatment arms; acetaminophen use did not differ between the two patient groups (Table 4), and no more than 16% of patients used rescue medication at any time (Table 4).

**Table 3:** **Responder variables, weeks 18 and 26 (per-protocol set)**

**Visit (week)**	**Artz**	**Durolane**	**Odds ratio (95%CI)**
	**(5 × 2.5 ml)**	**(1 × 3 ml, 4 × sham)**	
	**(** ***n*** **= 158)**	**(** ***n*** **= 161)**	***P*** **value**
**OMERACT-OARSI**			
Week 18			
Responder	146 (92.4%)	152 (94.4%)	1.15 (0.63; 2.09)
Nonresponder	12 (7.6%)	9 (5.6%)	0.6487
Week 26			
Responder	148 (93.7%)	151 (93.8%)	1.12 (0.61; 2.05)
Nonresponder	10 (6.3%)	10 (6.2%)	0.7129
**WOMAC pain subscale**			
Week 18			
Responder	116 (73.4%)	124 (77.0%)	0.91 (0.62; 1.35)
Nonresponder	42 (26.6%)	37 (23.0%)	0.6473
Week 26			
Responder	129 (81.6%)	127 (78.9%)	0.96 (0.65; 1.41)
Nonresponder	29 (18.4%)	34 (21.1%)	0.8157
**WOMAC pain ‘walking on a flat surface’ item**			
Week 18			
Responder	152 (96.2%)	149 (92.5%)	2.12 (1.14; 3.94)
Nonresponder	6 (3.8%)	12 (7.5%)	0.0176
Week 26			
Responder	153 (96.8%)	148 (91.9%)	2.26 (1.23; 4.12)
Nonresponder	5 (3.2%)	13 (8.1%)	0.0082

Data presented as *n* (%). Odds ratio obtained from a generalized estimable equation model. Durolane from Q-med AB (Sweden) and Artz from Seikagaku Corporation (Japan). CI, confidence interval; OMERACT-OARSI, Outcome Measures in Rheumatoid Arthritis Clinical Trials – Osteoarthritis Research Society International; WOMAC, Western Ontario and McMaster University.

**Table 4:** **Summary of rescue medication use-total grams used by visit (per-protocol set)**

**Time interval**	**Statistic**	**Artz**	**Durolane**
		**(5 × 2.5 ml)**	**(1 × 3 ml, 4 × sham)**
		**(** ***n*** **= 158)**	**(** ***n*** **= 161)**
Pre treatment	*n* (%)	2 (1.3%)	4 (2.5%)
	Mean (SD)	1.25 (0.35)	1.25 (0.87)
	Median	1.25	1.00
Week 4 to week 6	*n* (%)	20 (12.7%)	26 (16.1%)
	Mean (SD)	4.48 (5.81)	4.75 (5.41)
	Median	2.00	2.75
Week 6 to week 10	*n* (%)	13 (8.2%)	14 (8.7%)
	Mean (SD)	3.12 (3.90)	4.14 (6.13)
	Median	1.50	1.50
Week 10 to week 14	*n* (%)	10 (6.3%)	6 (3.7%)
	Mean (SD)	2.75 (3.83)	3.58 (3.76)
	Median	1.00	2.25
Week 14 to week 18	*n* (%)	9 (5.7%)	5 (3.1%)
	Mean (SD)	4.56 (6.05)	2.60 (2.30)
	Median	1.50	2.50
Week 18 to week 26	*n* (%)	21 (13.3%)	17 (10.6%)
	Mean (SD)	6.10 (6.28)	6.26 (6.94)
	Median	3.50	3.50

Only patients that used rescue medication were included. Durolane from Q-med AB (Sweden) and Artz from Seikagaku Corporation (Japan). SD, standard deviation.

When efficacy analyses were repeated using the ITT set, all conclusions were identical. In the subgroup analyses (gender, age, and Kellgren–Lawrence grade II and III at baseline) there were no statistically detectable or clinically relevant differences.

The incidence of AEs was similar in patients receiving Durolane or Artz (Table 5). The three most common TEAEs were arthralgia (14.9% and 16.7%, for Durolane and Artz respectively), upper respiratory tract infection (7.4% and 4.6%), and injection site pain (4.0% and 3.4%). Among nine patients reporting serious AEs, none were judged to be treatment related. TRAEs affected 13.1% of Durolane patients and 9.8% of Artz patients. The most common TRAE was arthralgia followed by joint swelling, and TRAEs related to the study knee were balanced between study arms. Severity in both groups was mainly mild to moderate (one injection site pain in the Durolane group was considered severe; two cases of arthralgia and one case of joint swelling in the Artz group were considered severe). The incidence of serious AEs was 1.7% (3/175) and 3.4% (6/174) in the Durolane and Artz groups, respectively, and no serious AE was related to the investigational products, as judged by the investigators. No death occurred in this study. Vital signs, blood chemistry and urine laboratories, and electrocardiogram results yielded no clinically relevant safety outcomes or treatment group differences.

**Table 5:** **Summary of treatment-emergent/treatment-related adverse events (safety set)**

**Adverse event category**	**Artz**	**Durolane**
	**(5 × 2.5 ml)**	**(1 × 3 ml, 4 × sham)**
	**(** ***n*** **= 174)**	**(** ***n*** **= 175)**
Patients with at least one treatment-emergent adverse event	74 (42.5%)	83 (47.4%)
Treatment-related adverse event	17 (9.8%)	23 (13.1%)
Treatment adjustment	1 (0.6%)	0
Treatment permanent stop	1 (0.6%)	0
Important^a^	2 (1.1%)	0
Severe	8 (4.6%)	6 (3.4%)
Serious^b^	6 (3.4%)	3 (1.7%)
All treatment-related adverse events^c^	17 (9.8%)	23 (13.1%)
Musculoskeletal and connective tissue disorders	16 (9.2%)	18 (10.3%)
Arthralgia	13 (7.5%)	15 (8.6%)
Joint swelling	3 (1.7%)	3 (1.7%)
Arthropathy	0	1 (0.6%)
Epicondylitis	1 (0.6%)	0
Joint effusion	0	1 (0.6%)
Limb discomfort	1 (0.6%)	0
Muscular weakness	0	1 (0.6%)
Musculoskeletal discomfort	0	1 (0.6%)
Myalgia	1 (0.6%)	0
Pain in extremity	0	1 (0.6%)
Skin and subcutaneous tissue disorders	0	1 (0.6%)
Erythema	0	1 (0.6%)

Data presented as number (%). Patients who experienced more than one adverse event are counted once in each row. Durolane from Q-med AB (Sweden) and Artz from Seikagaku Corporation (Japan). ^a^An important adverse event is any nonsevere adverse event leading to dose adjustment, interruption, or permanent stop. ^b^None of the serious adverse events were reported to be treatment related. ^c^Treatment-related adverse events were judged to have a definite, possible, or uncertain relationship to treatment.

## Discussion

We report a non-inferiority trial of Durolane (one injection) versus Artz (five injections) over 18 and 26 weeks. Patients were well matched at baseline (Table 1) and study retention was excellent (Figure 1). Both treatment groups showed a clinically significant, identical, and robust response to treatment, for the primary aim of pain (Table 2), for secondary aims of physical function, global self-assessment, and knee stiffness (Table 2), and for exploratory responder variables (Table 3). Use of rescue medications was low (Table 4), and both Durolane and Artz are associated with few TRAEs (Table 5). Our results provide rigorous evidence that Durolane is non-inferior to Artz; both Artz and Durolane are safe, efficacious, and well tolerated.

Patients responded comparably well to Durolane and Artz (Table 3). However, there were statistically more nonresponders at weeks 18 and 26 with Durolane in the WOMAC pain ‘walking on a flat surface’ item, although there were few nonresponders overall (Table 3). Conversely, there were numerically more nonresponders with Artz according to the OMERACT-OARSI criteria, although this difference did not attain statistical significance (Table 3). This discrepancy between measures may be a function of the small number of nonresponders and a large sample size.

Use of rescue medication by patients was quite low overall (Table 4). Only 14% of patients used rescue medication within 4 to 6 weeks of treatment, and roughly 5% of patients used rescue medication during weeks 10 to 18 (Table 4). Low use of rescue medication is consistent with the high perceived efficacy of treatment in reducing pain (Table 2), even at weeks 18 and 26. After week 18, use of rescue medication began to increase, perhaps as a result of loss of efficacy of both products with time (Table 4). A similar study in Germany, which used Euflexxa versus Synvisc in the treatment of knee OA, reported that 49.3% of patients using Euflexxa and 81.9% of patients using Synvisc required rescue medication at some point during the trial [26].

Incidence of TEAEs was similar between the Durolane (47.5%) and Artz (42.5%) treatment groups (Table 5), consistent with similar knee HA trials [26,28-30]. A trial of different forms of HA reported that 37.1% of patients (119 of 321) experienced TEAEs, all of mild-to-moderate intensity [26], while a similar trial of two HAs reported that 55.0% of patients (326 of 588) experienced TEAEs [30]. TRAEs were less prevalent than TEAEs. Arthralgia was the common TRAE here (Table 5) and arthralgia is expected among patients who receive HA injections [29]. Our results suggest that a single Durolane injection for treatment of OA knee pain was safe and well tolerated.

The conclusion that Durolane is non-inferior to Artz is robust, as all four indications more than satisfied 8% non-inferiority criteria (Table 2). We report that the WOMAC pain response rate (Durolane + Artz) averaged 75.2% at week 18 and 80.3% at week 26 (Table 3), which is an unusually high response rate. In contrast, a RCT of HAs versus saline found that the WOMAC pain response rate to HA was 38.1% at week 13 and 36.3% at week 26 [28]. The OARSI response rates we report are also higher than is generally reported. For both Durolane and Artz, the OMERACT-OARSI response rate was at least 92% at weeks 18 and 26 (Table 3). One RCT of HAs versus steroid reported the OARSI response rate to HA was 63.3% at week 18 and 62.8% at week 26 [29]. The OARSI response rate to HA in a second RCT was 66% at week 12 and 67% at week 26 [30]. In a RCT with unusually long follow-up, the OARSI response rate to HAs ranged from 71.1% at 7 months to 80.5% at 40 months [31]. It is unclear how best to explain the strength of our results, although there are several possibilities.

It may be typical that Chinese patients respond well to HAs. To our knowledge, only four prior RCTs have been carried out in China to test HAs in treatment of knee OA, so we have no *a priori* expectations as to the robustness of expected responses. For example, HAs were compared with warm acupuncture [32] and with electroacupuncture [33], and both studies documented a good response to treatment. In the study of electroacupuncture [33], response to HA was comparable in magnitude with what we report; patients with stage II Kellgren–Lawrence scores showed a decline in total symptom scores from 12.2 to 6.5 over 5 weeks. Another Chinese study compared HA with glucosamine sulfate or to a combination of glucosamine sulfate and arthroscopic debridement [34], and this study found that all treatments were efficacious. A final study compared HA with meloxicam in adult patients with Kashin–Beck disease, a chronic osteochondropathy largely limited to China [35]. In this study, patient improvement from baseline in the WOMAC A (pain) score was 4.6 points over 12 weeks, among 80 patients who had HA (25 mg) injected into the target knee at weekly intervals for 3 weeks [35]. The WOMAC A score was 12.5 at baseline and 7.9 at week 12, for an average improvement of 4.6 points [35]. In our study, the WOMAC A score was 9.5 at baseline (Table 1) and improved by an average of 5.9 points over 18 weeks (Table 2). Hence, our results show roughly comparable pain relief in OA patients with those in patients with Kashin–Beck disease [35].

Most patients were naïve to any treatment before study enrollment (Table 1), and analgesic use in China is low overall compared with the United States [36]. It is therefore possible that patients benefitted from initiation of treatment or from encouragement to use analgesics for pain control. Nevertheless, analgesic use overall was quite low (Table 4). There can be ethnic differences in how efficiently acetaminophen is metabolized; such differences in susceptibility to pain alleviation [37] could potentially explain low use of analgesics in our study (Table 4). In addition, metabolism of acetaminophen is affected by gender, oral contraceptive use, and smoking [38] and such factors can differ from study to study. Acetaminophen glucuronidation is higher in males than in females, such that male smokers have the highest rate and female nonsmokers or noncontraceptive users have the lowest rate of glucuronidation [38].

Ethnic differences in pain sensitivity have been documented [39-43], and such differences could potentially result in clinically significant differences in reported pain. Little is known about whether documented experimental differences in the thermal pain threshold might influence patient willingness to rehabilitate aggressively or to abstain from use of rescue medications.

A criticism of our work is that we did not use an inactive placebo arm, so it is unclear how much of the documented efficacy (Tables 2, 3, and 4) can be attributed to a placebo response. Placebo arms have been recommended even in surgical RCTs, and improvement in the placebo arm was documented in 39 of 53 surgical RCTs [44]. The majority of past HA trials have used placebo – defined as saline injection or arthrocentesis of the joint space – and the conclusion is broadly that HAs are superior to placebo [45]. However, neither saline injection nor arthrocentesis is truly inactive [46], and subcutaneous placebos are known to be more effective than oral placebos [47]. The alternative to inactive placebo is to compare a new medication with the best current medication, accepting that such comparator trials may produce higher placebo response rates than placebo-controlled trials [48]. Comparator trials are ethically easier to defend than placebo-controlled trials because they provide treatment for more patients [48]. Further, because superiority of active medication may be easier to achieve over inactive placebo than over competing medication, placebo-controlled trials may allow drugs into the marketplace that are less efficacious than those already available [49]. Our goal here was conservative; to test the hypothesis that pain reduction with Durolane was non-inferior to Artz, because Artz has a long record of safety and efficacy in China. A placebo arm would have increased costs substantially, while providing no greater clarity as to whether Durolane is comparable with the established treatment option.

The relative merits of one injection versus five injections are an important issue for physicians using HA to treat knee OA. Patients might prefer to have one injection, if they could be certain that one injection was as effective as five injections. Such a preference could be driven by considerations such as pain of multiple injections and inconvenience of multiple clinic visits. However, physicians might prefer to give five injections, for several reasons: multiple patient visits give the physician more opportunity to monitor the patient over time and to address AEs that might otherwise go unaddressed; and the adverse consequences of accidentally missing the joint space in a single injection are minimized if the patient is scheduled to receive additional injections. How these considerations influence physician choice of medications is a topic that should be addressed in future research.

## Conclusions

We found that both Durolane and ARTZ were effective: more than 90% of all patients reported a favorable response by OMERACT-OARSI criteria over time periods as long as 18 and 26 weeks (Table 3); at least 92% of patients had a decrease in pain while walking on a flat surface (Table 3); and more than 77% of patients reported a decrease in symptoms assessed by the WOMAC pain subscale (Table 3). These results demonstrate that one injection of Durolane and five injections of ARTZ are comparably safe, effective, and well-tolerated treatments for mild to moderate knee OA.

## Abbreviations

AE: adverse event
CFB: change from baseline
HA: hyaluronic acid
ITT: intention-to-treat
OA: osteoarthritis
OMERACT-OARSI: Outcome Measures in Rheumatoid Arthritis Clinical Trials – Osteoarthritis Research Society International
RCT: randomized clinical trial
TEAE: treatment-emergent adverse events
TRAE: treatment-related adverse events
WOMAC: Western Ontario and McMaster University
